# Valuing behavioral interventions for obesity reduction: A scoping review of economic models

**DOI:** 10.1111/obr.13865

**Published:** 2024-11-17

**Authors:** Joanna McLaughlin, Carlos Sillero‐Rejon, Theresa H. M. Moore, Hugh McLeod

**Affiliations:** ^1^ Musculoskeletal Research Unit, Translational Health Sciences, Bristol Medical School, University of Bristol Southmead Hospital Bristol UK; ^2^ Population Health Sciences, Bristol Medical School University of Bristol Bristol UK; ^3^ National Institute for Health and Care Research Applied Research Collaboration West (NIHR ARC West) University Hospitals Bristol and Weston NHS Foundation Trust Bristol UK

**Keywords:** behavioral intervention, health economics, modeling, obesity

## Abstract

Policymakers require health economic modeling to guide their decision‐making over the choice of interventions for obesity. This scoping review was undertaken to report on the health economic models in use for estimating the value of behavioral interventions (individual or population level) for obesity reduction. Electronic databases (MEDLINE, Embase, PsycINFO, EconLit, and Web of Science) were searched for publications meeting inclusion criteria from January 2015 to May 2023. Seventy‐three studies were included, using 44 health economic models between them. When considered against the expert recommendations for modeling of this type, only four models (9%) met all five key elements. The element most commonly unfulfilled was the use of a microsimulation modeling approach (41%, *n* = 18), followed by model validation (46%, *n* = 20). A majority of models met each of the other elements: use of a lifetime horizon (59%, *n* = 26), inclusion of key health events (66%, *n* = 29), and a risk equation approach to event simulation (71%, *n* = 31). In addition, under half of the studies considered health inequalities in their reporting. Continued proliferation of models with inadequate time horizons, breadth of obesity‐related health conditions, and perspectives on costs and outcomes risks underestimation of the benefits of longer term interventions and impacts on health inequalities.

**Abbreviations**
BMI: body mass index
CVD: cardiovascular disease
NICE: National Institute for Health and Care Excellence
OECD: Organisation for Economic Co‐operation and Development
QALYs: quality‐adjusted life years

## 1 INTRODUCTION

### 1.1 Health economics in healthcare decision‐making

Policymakers concerned with obesity and its impact on health need to make decisions about the provision of interventions for the management and prevention of overweight and obesity and rely on economic evaluation to estimate their value for money.^1^ In economic evaluation, cost‐effectiveness analyses are the preferred methodology to evaluate the effects and consequences of alternative interventions relative to their cost.^2^ A cost‐utility analysis is a type of cost‐effectiveness analysis where the health outcomes are measured using quality‐adjusted life years (QALYs). QALYs are a measure of health‐related quality of life over time: One QALY is 1 year of life lived in perfect health. A year lived in various states of health can be quantified on a scale in which zero represents death and one represents perfect health.^3^ Cost‐utility analysis and, therefore, QALYs are the main methods used by the National Institute for Health and Care Excellence (NICE) in the United Kingdom to determine the cost‐effectiveness of public health interventions.^4^ Interventions must generally cost ≤£30,000 per QALY gained to be deemed acceptably cost‐effective for use in the NHS by NICE. In these calculations, NICE accounts for costs and benefits to health and social care; however, public health and wider economic perspectives on obesity recognize that its costs and impacts extend far beyond these spheres.^1^

### 1.2 The role of health economic modeling

Health economic modeling is a technique that fulfills several functions including the need to extrapolate measured short‐term cost and outcome data from trials of interventions in specific settings, to longer timeframes of anticipated treatment effects and associated costs, for the purpose of estimating cost‐effectiveness.^3^ A taxonomy of models used for economic evaluation has been described reflecting the variation in model and intervention complexities; each type offers advantages and disadvantages.^5^, ^6^ Microsimulation modeling refers to the use of computational models capable of simulating the trajectories of high numbers of single individuals over time through multiple health states with complex interactions between the individual's characteristics and their changing health states. A list of other model types and their descriptions are provided in Table S1. Uncertainty is inherent in health economic modeling—decisions must be made over the sources of data used to create the model (judgment uncertainty), and statistical uncertainty results from the decisions made in the choice of a model's structure and parameters.^7^

Key elements in any economic evaluation and, therefore, in health economic modeling are those of the choice of perspective and of the time horizon to be modeled. Perspective is the lens through which the costs and benefits of an intervention are considered, for example, healthcare payers or societal. Time horizons within modeling are the length of time over which an intervention's costs and benefits are assessed.

### 1.3 Health economic modeling in obesity

Modeling for obesity raises particular complexities.^8^ In determining the effect of an intervention on body mass index (BMI), information on outcomes may be available directly from trials of the intervention, or assumptions may need to be made regarding changes in intermediary factors such as calorie intake and physical activity levels. Decisions must also be made over how BMI will be assumed to change over time after an intervention is complete. In NICE's analyses, the rate of weight regain was identified as the most important factor in the cost‐effectiveness of weight management interventions.^9^ Interventions to prevent obesity or to support weight management in those already living with obesity are often behavioral in nature, for example, dietary modification, increased physical activity, and behavioral therapy. These may be introduced at an individual or population level.^10^ This review considers the modeling of behavioral interventions for obesity in recognition of the prominence of these interventions in obesity policymaking, and the relevance of the complexities described above in quantifying sustained BMI changes.^1^

The impact of a change in BMI on health is equally complex. Modelers must make decisions over the breadth of clinical conditions and risk factors to include in their consideration of the impact of a change in BMI on a person's health, balancing practicality (e.g., data availability) with completeness.^11^ BMI itself is acknowledged as an imperfect measure of obesity and its sequelae, and models may need to use alternative measures of weight and body composition changes.^12^

Schwander et al.'s 2016 systematic review of obesity intervention economic modeling identified a high number of published models (*n* = 72) concluding that overall consensus is lacking about the most appropriate approach.^8^ This variation may undermine confidence in current estimates of intervention cost‐effectiveness and the development of future interventions. Recommendations following an expert panel rating of key elements in health economic obesity models were published in 2020.^13^ These are detailed in Table S2. In brief, they require that models should use a risk equation approach (the risk is calculated as an equation of risk factors, and the intervention effect is simulated by the change of those risk factors), microsimulations were preferred, both short‐term and long‐term effects should be presented with a lifetime horizon being optimal, and models should be internally and externally validated. There was no consensus on the epidemiological events that should be included; however, there was a minimum consensus on including mortality, cardiovascular disease (CVD), and diabetes.

Comprehensive, valid modeling of obesity is necessary for decision‐makers seeking to determine and then justify effective policy responses to obesity as a public health issue. Inadequacies in current modeling have been highlighted as a priority for action in improving obesity policy response in the United Kingdom.^1^ This scoping review aimed to identify published models used to evaluate the long‐term cost‐effectiveness of behavioral interventions to reduce overweight and obesity since the 2016 systematic review, to analyze the nature and variation of the models, and to determine whether they meet expert recommendations.

## 2 METHODS

The methodology for the scoping review was based on guidelines from the Joanna Briggs Institute,^14^ and the reporting was guided by the Preferred Reporting Items for Systematic reviews and Meta‐Analyses (PRISMA) extension for scoping reviews.^15^ The specific terms for the search strategy and inclusion criteria were informed by previous, related systematic reviews.^8^, ^16^ The study protocol was documented in advance of the data collection and was registered on the Open Science Framework platform.^17^

We conducted this scoping review in two stages. In the first stage, publications using relevant health economic models were identified to allow reporting on the frequency and nature of model usage. In the second stage, data on the models themselves were retrieved from the publications to allow reporting on the content of the models and their comparison against the existing expert recommendations.

### 2.1 Stage 1—identifying recent publications using relevant modeling

#### 2.1.1 Sources of evidence and eligibility criteria

A systematic search was conducted for recent studies meeting predetermined eligibility criteria that have applied or described economic modeling to evaluate behavioral interventions for the reduction of overweight and obesity. The inclusion and exclusion criteria are detailed in Table 1.

**TABLE 1:** Inclusion and exclusion criteria.

	Inclusion criteria	Exclusion criteria
Population	● General population of adults, which will include people living with overweight or obesity ● Clinical populations receiving treatment for obesity or overweight	● Populations limited to specific comorbidities, for example, pregnant women with obesity ● Populations of children alone, or studies on family interventions, which report only on the children's outcomes ● Populations outside the Organisation for Economic Co‐operation and Development (OECD)
Intervention	● Interventions or policies targeting behavioral risk factors (e.g., calorie intake, sugar intake) for preventing or reducing obesity and/or overweight ● Hypothetical scenarios where level of obesity in the general population is altered without consideration of a specific intervention	● Interventions or policies aimed only at altering physical activity or improving nutrition where weight management is not the primary intent ● Evaluations of medication/surgery interventions without “usual care” or behavior change interventions as a comparator
Comparator	● No intervention or policy ● Current practice ● Hypothetical simulation	
Main outcomes	● The incremental health effects (e.g., utilities) and cost for a time horizon of at least 10 years ● Perspective that includes healthcare	● Short‐term time horizons alone (<10 years) ● Uncosted health outcomes only
Study types	● Any study type incorporating economic modeling of cost‐effectiveness of interventions/policies for the management or prevention of overweight and/or obesity ● Publication on or after January 1, 2015	● Studies where the health outcomes and cost are calculated through methods other than economic models ● Reviews, editorials, commentaries, protocols, and methodological articles

The searches were performed within the MEDLINE, Embase, PsycINFO, EconLit, and Web of Science databases from 2015, when the last similar review search finished,^8^ to May 2023. The electronic search strategy is detailed in Table S3; we included terms related to obesity and weight management and terms for heath economic modeling and limited the search to studies in English language. Hand searches were also made of the citations within the included references.

#### 2.1.2 Data extraction

The titles and abstracts of the references were screened by two reviewers blinded to each other's decisions. References that evidently did not meet inclusion criteria were excluded. Full‐text articles for all remaining references were examined to determine whether they met the inclusion and exclusion criteria. Where review articles were returned by the search, the citations included in the reviews were checked for relevant studies to be screened for inclusion. A bespoke electronic form was developed, piloted, and then used to extract data from the full‐text document for each included study.

### 2.2 Stage 2—examining the models' content

#### 2.2.1 Sources of evidence

The primary source of each model identified in the references from Stage 1 was identified and retrieved where possible, including relevant supplementary material and technical documents. If the primary source was unavailable, the most recent and complete reference describing the model was retrieved.

#### 2.2.2 Data extraction

Data were extracted using a bespoke electronic form by two reviewers independently. The following data were extracted: model name, year, author, relevance to weight loss/obesity reduction, model type (e.g., microsimulation), model population inputs, behavioral risk factors modeled (e.g., dietary change), model intervention inputs (e.g., weight loss effect, intervention costs), health effects and events modeled, event simulation approach, costs, utilities (e.g., QALYs), discount rate, economic perspective, time horizons, and validation. Table S1 contains the full explanation of the categorization and definitions of model types. Event simulation approaches were categorized as (1) risk equation (base risk is calculated as an equation of risk factors, and the intervention effect is simulated by the change of risk factors); (2) BMI‐related relative risk of disease incidence (an incidence estimate [e.g., age‐specific] is used as base risk, and the intervention effect is simulated by applying a relative risk for BMI or obesity status to the base risk); and (3) BMI function (base risk is calculated as a function of the baseline BMI, which is then directly influenced by the intervention effect on the BMI).

#### 2.2.3 Synthesis

Descriptive summary statistics, illustrated in tables or figures, were produced for each of the main categories of data extraction using the statistical software STATA 17.^18^

Where models were used across multiple included studies, with variation in the way in which they had been used, variables were represented in the affirmative if any of the studies had used that variable in their modeling. For example, where a model is indicated to have used several health events as outcomes, not all of these health events may have been used in all the included studies, which used the model.

An overview of the models was created and considered against the relevant expert panel recommendations published by Schwander et al.^13^ regarding the nature and content of models relating to obesity reduction. The recommendations are clear on four of the elements: A lifetime time horizon should be used, microsimulation is the preferred model type, risk equations are the preferred event simulation approach, and the model should be validated. Less clarity was possible over the health events to be included in the model; models were considered to meet this element of the recommendations if they included mortality, CVD, and diabetes as a minimum. Additionally, data were extracted to allow reporting of the prevalence of the models' inclusion of health inequalities considerations in their outputs. Though this is not one of the existing expert recommendation elements, a need to address health inequalities in modeling is prominent in health economics and obesity intervention literature.^19^, ^20^

## 3 RESULTS

### 3.1 Scoping review Stage 1 results—recent studies using relevant modeling

**FIGURE 1:**
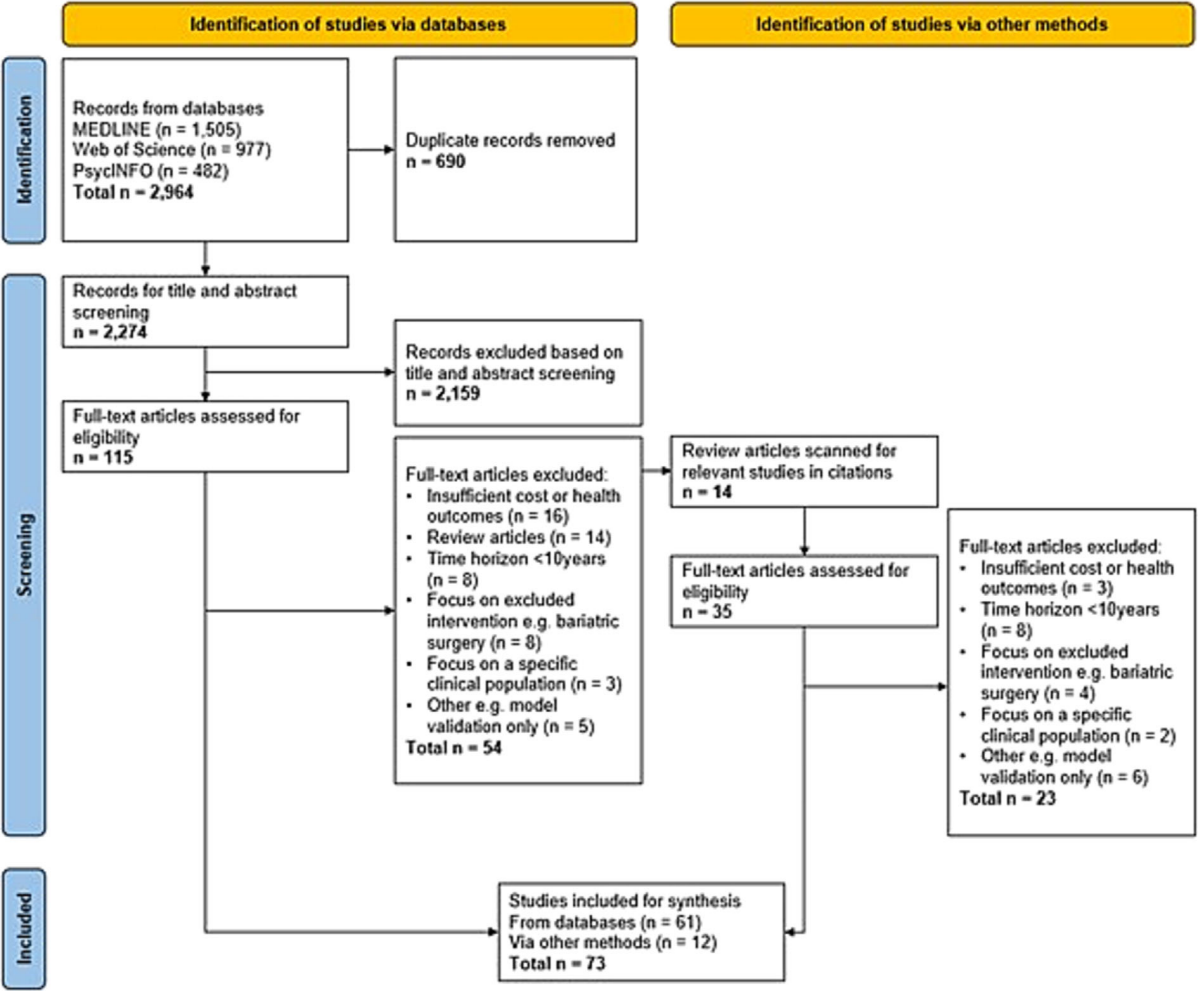
Preferred Reporting Items for Systematic reviews and Meta‐Analyses (PRISMA) flow chart.

Figure 1 displays the PRISMA flow chart detailing the search strategy output and the exclusions made from the results. After screening and full‐text articles assessment, 73 studies were included in the final selection. These are detailed in Table S4.

#### 3.1.1 When were they published and in which country?

A mean of nine studies were published in each year of the search timeframe. Most of the studies were based in North America (36%), followed by Australia or New Zealand (26%) and the United Kingdom (21%).

#### 3.1.2 What were they looking at?

Fifty‐eight of the studies (79%) assessed the impact of an intervention—actual or hypothetical. The remainder modeled the impact of existing (10%, *n* = 7) or changing (11%, *n* = 8) patterns of obesity among the population on health.

Table S5 details the range in the types of interventions modeled. Individual‐level interventions for weight management (26%, *n* = 15) or for more general health improvement (21%, *n* = 12)—commonly diabetes prevention programs—were mostly based on measured outcomes from trials or evaluations of existing interventions. Population‐level interventions regarding taxation, subsidies, or food industry regulation were mostly hypothetical in nature.

#### 3.1.3 What perspective was taken over what time horizon?

The majority of studies undertook modeling using a healthcare only perspective (60%, *n* = 44). Three studies (4%) considered social care in addition to healthcare. Thirteen studies (18%) used a societal perspective, and 11 studies (15%) used both a healthcare and a societal perspective (reported separately). In two studies (3%), the perspective taken was not clear.

A lifetime time horizon was used in 46 (60%) of studies. The shortest maximum time horizon used reflects the study inclusion criteria and was therefore 10 years (18%, *n* = 14). The remaining studies (22%, *n* = 17) used maximum time horizons of between 15 and 35 years.

#### 3.1.4 Were health inequalities examined?

In 34 (47%) of the studies, there was an examination of the differential effect of the impact of obesity and health effects on population subgroups, amounting to a consideration of health inequalities within the modeling. The most common stratification was by socioeconomic deprivation (*n* = 15), while others considered inequality by geography, race, age, and sex.

#### 3.1.5 How many different models were used and what was the broad nature of these models?

There were 44 separate models in use across the 73 studies. In 24 studies (33%), the authors had created a new model for the purposes of their study. Overall, 35 studies (48%) used a model developed by their own author group. Table S6 lists the models used in order of frequency, naming the 12 models, which were used multiple times: One model was used 10 times, and 32 models were only used once.

### 3.2 Scoping review Stage 2 results—nature of the models

This section presents the data extracted from the 44 models found to have been used across the 73 studies. The frequency of model use across the studies does not feature in the data presentation—all models are presented with equal weight. Table S7 provides the details of all the models included in the review.

#### 3.2.1 What were the model types?

Of the 44 models, 42 (96%) were categorized as state transition models (microsimulation [41%, *n* = 18], Markov [34%, *n* = 15], multistate life table [18%, *n* = 8], or comparative risk assessment models [2%, *n* = 1]). There was also one disease event simulation model (2%) and one decision tree model (2%).

There were differences in how the models estimated the change in incidence and prevalence of health events—their event simulation approach. Most models (68%, *n* = 30) used a risk equation approach, taking into account multiple risk factors and changes in these risk factors with alterations in BMI to calculate the likelihood of health events. The remaining models used much simpler approaches—either applying the relative risk of various health events to individuals based on their BMI (14%, *n* = 6) or calculating health outcomes as a direct function of an individual's BMI alone (14%, *n* = 6).

#### 3.2.2 What were the outputs and perspectives of the models?

QALYs were the primary health economic measure in 27 (61%) models. Other models used either a combination of health‐adjusted life years and/or disability‐adjusted life years (*n* = 6, 14%) or made direct cost calculations based on health status (*n* = 6, 14%) or BMI (*n* = 5, 11%).

Regarding the time horizons available within the models, 26 (59%) offered a lifetime time horizon, and nine (21%) also offered short‐term (<10 years) estimates. Where no lifetime horizon was offered, the maximum time horizon ranged from 10 to 35 years.

Use of a healthcare only perspective was most common (55%, *n* = 24). Most of the remainder took societal perspectives, either with (23%, *n* = 10) or without separate presentation of a healthcare perspective (16%, *n* = 7). The component outcomes used in the societal perspectives are further detailed in Section 3.2.5 below.

#### 3.2.3 What were the inputs of the models?

Three of the models (7%) did not provide information on the demographic and other baseline variables included in their models. Within the other 41 models, 40 (98%) used age, 39 (95%) used sex, and 17 (42%) used ethnicity as their demographic inputs. Thirteen models (32%) used a measure of deprivation or education level, 34 (83%) used baseline BMI or weight as an input variable, and 13 (32%) incorporated baseline comorbidities or health status.

#### 3.2.4 How was the interventions' effect on obesity status modeled?

In their approach to modeling the impact of obesity reduction, 39 models (89%) used BMI change from baseline. Only four models (9%) also included a measure of BMI change maintenance. The other models used change in obesity status from baseline (9%, *n* = 4) or examined the impact of current obesity prevalence without modeling any changes (2%, *n* = 1). Models examining interventions used changes in purchasing (*n* = 2), changes in calorie consumption (*n* = 16), and change in physical activity or use of active transport (*n* = 5) as applicable.

#### 3.2.5 Which health events and outcomes were modeled?

**FIGURE 2:**
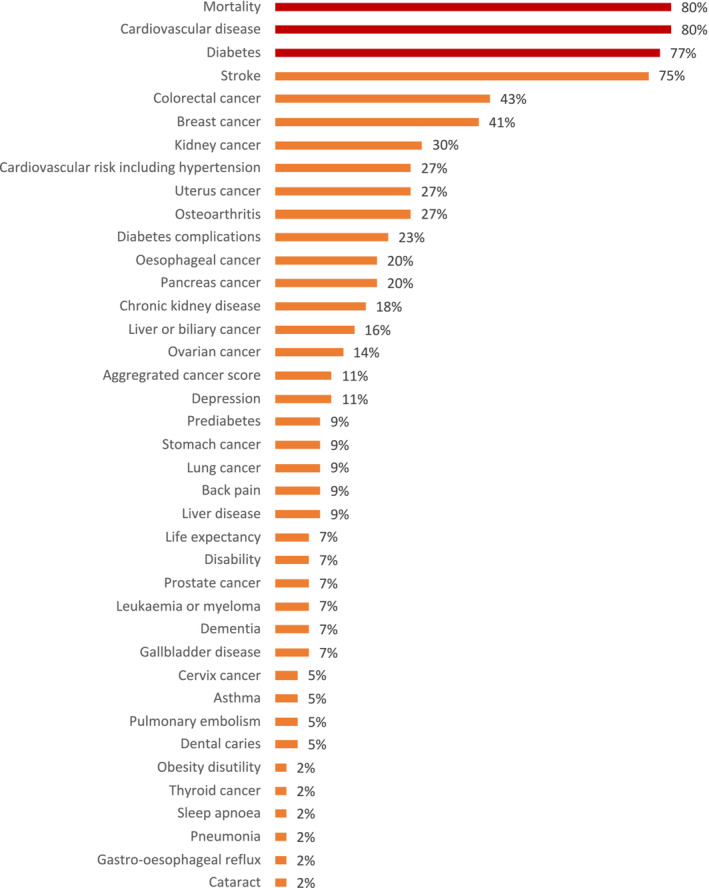
Percentage of models including each health event, *n* = 44.

Four models (9.1%) did not specify which health events they measured. Models that did specify the health events, which had been included, used up to 32 different health events (range 1 to 32, mean 8.5, SD 6.3). The percentage of models including each of the health events is presented in Figure 2. The most commonly included health events were mortality (*n* = 35, 80%), CVD (*n* = 35, 80%), diabetes (*n* = 34, 77%), and stroke (*n* = 33, 75%). Fifteen different types of cancer were included across the models, including aggregate variables of common cancers; the most commonly included were colorectal cancer (*n* = 19, 43%) and breast cancer (*n* = 18, 41%). Osteoarthritis was included in 12 models (27%). Depression was included in five models (11%).

Additional, non‐health consequences were included in the model output where a societal perspective was taken. In order of frequency, the additional measures were productivity (*n* = 14), tax (*n* = 5), use of informal care (*n* = 2), and single uses of income and health insurance.

Where described, models used published estimates of population attributable fractions of obesity for each health event, or individual observational studies reporting on incidence and prevalence of chronic conditions and health events in patients living with obesity. Other than in some microsimulation models, chronic conditions were considered to be independent of each other for the purposes of modeling. None of the models included differential costs for the severity of each condition, for example, differences in patients who do and do not undergo surgery for their osteoarthritis, though in the case of diabetes, some models included an additional consideration of diabetic complications (*n* = 10, 23%).

#### 3.2.6 Were the models validated?

Validation techniques or results were sparsely reported. There was often no further detail offered beyond a statement that validation had been undertaken, including a lack of specificity as to whether this was internal or external validation. Twenty (46%) of the models provided evidence of validation either alongside the published modeling outputs or in separate publications.

#### 3.2.7 How did the models compare to the expert recommendations?

**FIGURE 3:**
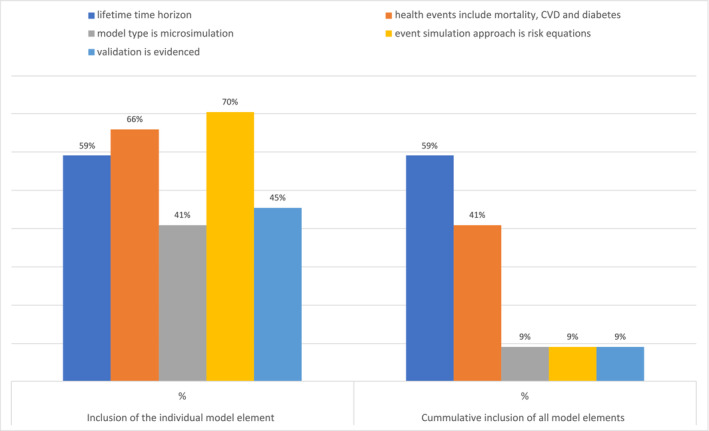
Percentage of models meeting elements of the expert recommendations, *n* = 44. CVD, cardiovascular disease.

Figure 3 displays the percentage of the models, which included each of the five model elements, that are in line with the expert recommendations. Only four of the models met all five elements: the CVD‐PREDICT model,^21^ Choi et al.'s model,^22^ the UK Health Forum model,^23^ and the School for Public Health Research Diabetes Prevention Model.^24^ Only 18 of the models (41%) met two or more of the elements. The element most commonly unfulfilled was the recommendation to use a microsimulation modeling approach (41%, *n* = 18), followed by the recommendation to provide validation of the model (46%, *n* = 20). A majority of models met each of the other elements: use of a lifetime horizon (59%, *n* = 26), inclusion of key health events (66%, *n* = 29) and a risk equation approach to event simulation (71%, *n* = 31).

## 4 DISCUSSION

### 4.1 Summary of the key findings

This scoping review of the health economic modeling of interventions for obesity reduction included 73 studies, across which 44 models were in use. Approximately a third of the studies created their own new model, and in studies making use of existing models, only six models were used more than twice overall, reflecting a lack of consensus on modeling approaches.

When considered against the expert recommendations for modeling of this type, only 18 models met two or more of the five key elements, and only four models met all five. These four models were not the most recently published—one was available as early as 2017. There was high variation in the health events included in the models. Around two thirds of the models included the core health events of mortality, CVD, and diabetes, but the inclusion of a wider range of health events was rare. Under half of the studies considered health inequalities in their reporting.

### 4.2 Strengths and limitations

A comprehensive search for studies across 8 years and all OECD countries over multiple databases was completed. The opportunity to draw on the methodology and findings of a previous, though non‐recent, systematic review also advantaged the validity of the review's approach. The diversity and high volume of economic models identified during the methodological design for this study meant that a scoping review was an appropriate methodological choice to meet the objectives to allow an exploration of the modeling landscape. As a result, the review does not extend to a systematic quality and risk of bias assessment of each individual model that would have been possible in a more mature research field. The demonstration of problematic diversity in modeling approaches, including continued re‐invention of models rather than re‐assessment and improvements to existing models, is a key finding of this scoping review.

The ability to draw on existing published expert recommendations for the modeling of obesity interventions provided rigor to this review's consideration of the nature of the models. The published recommendations have limitations, however, as they resulted from a single consensus gathering exercise and were not intended as comprehensive modeling methodological guidelines.^13^ Within these recommendations, four of the five offered clear measures; however, when considering the health events that a high‐quality model should include, there was no specified list of events, and therefore, the choice here of core events as a proxy measure for this recommendation was necessary. The choice of mortality, CVD, and diabetes is a very narrow selection given that existing literature can support the association of obesity with many other health events.^11^ However, only a third of the models examined in this review included even these three health measures; therefore, the choice of a higher number of health measures would have been overly limiting as a threshold to report against.

### 4.3 Relationship to existing literature

The problematic lack of consensus in modeling of obesity, and the trend for author groups to design their own new models rather than use or modify one of the multitude of existing models, is evidenced here in this review to have continued since observations to this effect were noted by Schwander et al. in their systematic review in 2016.^8^ Despite their subsequent publication of expert recommendations for modeling obesity,^13^ this review has demonstrated that a majority of models in use in recent years fail to meet these recommendations.

Analogous review studies have been published in the field of childhood obesity and make recommendations aligned with the findings of this scoping review^16^, ^25^; considerations of weight loss maintenance are lacking in modeling, and wider sets of outcomes are not well measured in economic evaluations of obesity interventions. A 2021 scoping review of simulation modeling for dietary interventions also reported a lack of adequate model validation and of considerations of health inequalities in policy modeling.^20^

Tremmel et al.'s systematic review of cost‐of‐illness studies for obesity concluded that variation in the obesity‐related diseases included in the studies they found, along with inadequate inclusion of wider costs related to productivity and informal care use, also highlights issues with consensus in modeling in this field.^26^

### 4.4 Implications for policy, practice, and research

The UK's Institute for Government recently reported on the repercussions of high obesity rates and concluded that obesity policy has failed to redress obesity prevalence and the inequalities within it. They specifically address the role of health economic modeling in responding to this policy issue and state that the government should “improve different types of evidence […] produce larger, more sophisticated and more robust models.”^1^

It is simpler to implement time‐limited individual‐level interventions and model associated short‐term proxy outcomes, compared to population‐wide structural/environmental‐level policies, which may generate the required long‐term health benefits.^1^ However, to justify the higher investment required for a holistic approach, decision‐makers need assurance about value for money. This is reliant on the availability of suitable economic models that can give confidence in the long‐term value of different interventions.

Addressing the issue of inappropriate, heroic assumptions about the long‐term impact of short‐term reductions in BMI would reduce current overestimates of the ease of achieving the behavior change required to cause and maintain weight loss. This approach can give policymakers unwarranted reassurance that investment in these types of interventions is sufficient. Improvements in the rigor of model validation and its reporting would drive accuracy in modeled outputs, further improving the ability of policymakers to select evidence‐based approaches to obesity reduction.

Current models tend to only include the major obesity‐associated outcomes: cardiovascular disease, cerebrovascular disease, some cancers, and type 2 diabetes. When a Mendelian randomization approach used 240 health‐related outcomes instead of just the main four, the effect of a BMI change on QALYs was around 300% greater than typically predicted. The authors concluded that previous cost‐effectiveness studies have likely underestimated the effect of BMI on quality of life and, therefore, the potential cost‐effectiveness of BMI interventions.^11^ The impacts of BMI on mental health, musculoskeletal health, and productivity are just three of the major factors often missing from current models.

To tackle health inequalities alongside the choice of interventions that offer good value, models must move away from one‐size‐fits‐all assumptions in intervention effects and the utility of resultant health states for different groups. This lack of nuance hinders the understanding of which interventions contribute most to addressing health inequalities. Policymakers now seek this information for informed health spending decisions.

## 5 CONCLUSION

Examination of the current landscape of health economic modeling in interventions and policies for obesity reduction and prevention in this review supports the need for improved modeling approaches and data availability to better address the design and commissioning of interventions for obesity. Health economic modeling, while not a solution in itself, is certainly necessary for improved policy decision‐making, and the current lack of consensus in modeling approaches and the availability of models meeting existing expert recommendations hinder progress at present.

Collaborative efforts between researchers, clinicians, public health professionals, and policymakers can pave the way for more effective obesity policy response, by improving the recognition of the potential value of interventions with long‐term impact including on health inequalities.

## CONFLICT OF INTEREST STATEMENT

None declared.

## Supporting information


**Table S1.** Definitions of ‘model type’ categories used in data extraction – reproduced from study protocol ^1^.
**Table S2.** Overview of key expert recommendations for health economic obesity models modified from Schwander et al 2020 ^2^.
**Table S3.** Search strategy terms for the health economic scoping review.
**Table S4.** Table of studies included in the scoping review.
**Table S5.** Actual and hypothetical interventions modelled.
**Table S6.** Frequency of model use.
**Table S7.** Table of the models included in the scoping review.
