# Supplementary material for: Valuing behavioral interventions for obesity reduction: A scoping review of economic models
Source: Obes Rev. 2024 Nov 17;26(3):e13865. doi: 10.1111/obr.13865 (PMC11791378; doi:10.1111/obr.13865)
Supplement: Supplementary file 1 — Table S1. Definitions of ‘model type’ categories used in data extraction – reproduced from study protocol 1. Table S2. Overview of key expert recommendations for health economic obesity models modified from Schwander et al 2020 2. Table S3. Search strategy terms for the health economic scoping review. Table S4. Table of studies included in the scoping review. Table S5. Actual and hypothetical interventions modelled. Table S6. Frequency of model use. Table S7. Table of the models included in the scoping review. [file OBR-26-e13865-s001.pdf]

Supporting Information

Title:

Valuing behavioural interventions for obesity reduction: A scoping review of economic models

Authors:

Joanna McLaughlin*[1] (ORCID id: 0000-0001-9921-4698)

Carlos Sillero-Rejon[2,3]

Theresa Moore[2,3]

Hugh McLeod[2,3]

[1]Musculoskeletal Research Unit, Translational Health Sciences, Bristol Medical School, University of Bristol, Learning and Research Building, Level 1, Southmead Hospital, Bristol, BS10 5NB, United Kingdom

[2]Population Health Sciences, Bristol Medical School, University of Bristol, United Kingdom

[3]National Institute for Health and Care Research Applied Research Collaboration West (NIHR ARC West), University Hospitals Bristol and Weston NHS Foundation Trust, United Kingdom

*Corresponding Author. Contact details: Joanna.McLaughlin@bristol.ac.uk +44 117 456 0758

Table S1: Definitions of 'model type' categories used in data extraction – reproduced from study protocol [1]

Model Type	Definition
**Decision tree**	Simulate possible decisions and outcomes using branches to represent each potential event.
**Comparative risk assessment (CRA)**	These are commonly aggregate-level models that use population-attributable fractions to estimate how parameters describing the relationship between a risk factor and disease outcome would change following an intervention. Individuals can be simulated when combined with microsimulation.
**Markov models without interaction**	Markov models simulate how a population or individual moves between predefined health or disease states at a specific time interval (for example, annually). This incorporates a time component and allows for modelled populations to remain in a health state from one time interval to the next, and to loop back from a diseased state to a healthy state (recur), all based on a given transition probability.
**System dynamics models**	System dynamics models allow for populations to interact both with each other and with their environment. The probabilities of events occurring in the model (the system) change through feedback as the model runs, governed by algebraic or differential equations.
**Markov chain models and Markov individual event history models**	In discrete or continuous time Markov chain models, state transition probabilities can depend on (interact with) the proportion of different populations in different disease states, and on the time that has elapsed in the model.
**Discrete event simulation (DES)**	Discrete event simulation (DES) is an extremely flexible modelling structure that simulates a system changing over time with a sequence of discrete individual events. Rather than simulating populations or individuals through states for a fixed length of time, multiple future events are in competition and the model jumps to whichever event occurs next based on predefined probabilities.
**Agent-based simulation (ABS)**	ABS models apply rules to agents or groups of agents, and responses depend on individual agent characteristics which can change either over time or following interactions with other agents or the environment. This is compared to system-based rules found in DES
**Multistate life tables**	Use of multistate life tables can be made with decision tree, comparative risk assessment, and Markov models with no interaction. Multistate life tables are defined as life tables that model an individual's, or proportion of a population's, probability of developing a given disease at different ages and subsequent case fatality rates once the disease is acquired. These can simulate multiple diseases simultaneously and can be used to add a temporal component to decision tree or CRA models.

**Microsimulation**	Use of microsimulation can be made with individual-level decision tree, comparative risk assessment, and Markov models.<br>In order to overcome the complexity of modelling multiple diseases and heterogeneous populations in decision tree, CRA, and Markov model structures, an alternative approach is to use individual patient simulation models (microsimulation). These allow for a population of heterogeneous individuals to move through the model based on probabilities appropriate to their characteristics (such as demographic factors or physiological characteristics). The model is run at the individual level with all members, or randomly selected members of a predefined population, being simulated until either a prespecified outcome occurs or a certain length of time has elapsed (e.g., death or reaching age 100).

Table S2: Overview of key expert recommendations for health economic obesity models modified from Schwander et al. 2020 [2]

Model aspect	Expert panel recommendations
**Time horizon**	Lifetime time horizon is optimal<br>Both short and long-term results should be presented
**Obesity associated events**	No consensus was reached on which clinical events to include. Events with a strong association with obesity and a clear causal relationship to obesity should be included.
**Model type**	Microsimulation (individual patient) models were the preferred approach.
**Event simulation approach**	Risk equation approaches were preferred for simulating clinical events.
**External validation**	External validation is important.

Table S3: Search strategy terms for the health economic scoping review

	**OVID MEDLINE(R) ALL <1946 TO CURRENT>**
**1**	body weight changes/ or weight gain/ or weight loss/
**2**	("body fat" or overweight or over-weight or obes* or adiposity or "body composition" or weight or BMI or "body mass index").tw,kf.
**3**	1 or 2
**4**	exp models, economic/
**5**	*models, theoretical/
**6**	*models, organizational/
**7**	markov chains/
**8**	exp decision theory/
**9**	(markov* or monte carlo).tw,kf.
**10**	econom* model*.tw,kf.
**11**	(decision* adj2 (tree* or analy* or model*)).tw,kf.
**12**	(microsimulation? or micro-simulation?).tw,kf.
**13**	discrete event? simulation?.tw,kf.
**14**	or/4-13
**15**	3 and 14
**16**	limit 15 to (english language and humans and yr="2015 -Current")

Table S4: Table of studies included in the scoping review

First author	Year	Location of study	Scenario	Aim	Intervention	Model used	Reference
Ahern	2022	UK	intervention	models individual weight management intervention	weight loss programme	School for Public Health Research Diabetes Prevention Model.	[3]
Ahern	2017	UK	intervention	models individual weight management intervention	weight loss programme	UK Health Forum	[4]
Amies-Cull	2019	UK	hypothetical change	models population obesity reduction impact	sugar reduction	PRIMEtime-CE	[5]
An	2022	N America	hypothetical intervention	models population obesity reduction impact	food labelling	Au	[6]
Ananthapavan	2020	Australia/New Zealand	intervention	models individual weight management intervention	health improvement/prevention intervention	ACE-Obesity Policy model	[7]
Ananthapavan	2022	Australia/New Zealand	hypothetical intervention	models population obesity reduction impact	food labelling	ACE-Obesity Policy model	[8]
Ananthapavan	2022	Australia/New Zealand	intervention	models individual weight management intervention	health improvement/prevention intervention	ACE-Obesity Policy model	[9]
Arrospide	2022	Europe (non-UK)	no change, no intervention	models population obesity impact	models impact of obesity	Arrospide	[10]
Avenell	2018	UK	intervention	models individual weight management intervention	weight loss programme	UK Health Forum	[11]
Bastro-Abreu	2019	Other	intervention	models population obesity reduction impact	SSB tax	CHOICES (Childhood Obesity Intervention Cost-Effectiveness Study) model	[12]

Basu	2020	N America	hypothetical intervention	models population obesity reduction impact	SSB ban	Basu	13
Bates	2022	UK	hypothetical change	models population obesity impact	models impact of obesity	School for Public Health Research Diabetes Prevention Model.	14
Bjornelv	2021	Europe (non-UK)	no change, no intervention	models population obesity impact	models impact of obesity	Bjornelv	15
Blakely	2020	Australia/New Zealand	hypothetical intervention	models population obesity reduction impact	dietary taxes and subsidies	Blakely	16
Bourke	2018	Other	hypothetical intervention	models population obesity reduction impact	SSB tax	ACE-Prevention	17
Boyers	2021	UK	intervention	models individual weight management intervention	weight loss programme	UK Health Forum	18
Breeze	2017	UK	hypothetical intervention	models population obesity reduction impact	multiple	School for Public Health Research Diabetes Prevention Model.	19
Brown	2017	Australia/New Zealand	hypothetical intervention	models population obesity reduction impact	active transport	Brown	20
Brown	2017	Australia/New Zealand	hypothetical intervention	models population obesity reduction impact	fuel tax	Brown	21
Brown	2017	Australia/New Zealand	hypothetical intervention	models population obesity reduction impact	active transport	ACE-Obesity Policy model	22
Chen	2016	N America	intervention	models individual weight management intervention	health improvement/prevention intervention	Chen	23
Chen	2022	N America	intervention	models individual weight management intervention	health improvement/prevention intervention	Chen	24

Choi	2017	N America	hypothetical intervention	models population obesity reduction impact	food subsidy	Choi	[25]
Cleghorn	2019	Australia/New Zealand	hypothetical intervention	models individual weight management intervention	weight loss programme	BODE (Burden of Disease Epidemiology, Equity and Cost-Effectiveness) Programme model	[26]
Cobiac	2017	Australia/New Zealand	hypothetical intervention	models population obesity reduction impact	dietary taxes and subsidies	ACE-Obesity Policy model	[27]
Crino	2017	Australia/New Zealand	hypothetical intervention	models population obesity reduction impact	SSB reformulation	ACE-Obesity Policy model	[28]
Dall	2015	N America	intervention	models individual weight management intervention	health improvement/prevention intervention	Dall	[29]
Fallah-Fini	2017	N America	no change, no intervention	models population obesity impact	models impact of obesity	Fallah-Fini	[30]
Galvain	2021	UK	usual care	models individual weight management intervention	usual care	Galvain	[31]
Goryakin	2019	Europe (non-UK)	hypothetical intervention	models population obesity reduction impact	active transport	OECD SPHeP-NCD (Strategic Public Health Planning for NCDs) model	[32]
Gray	2018	UK	intervention	models individual weight management intervention	weight loss programme	The Cardiovascular Disease (BMI) Policy model	[33]
Gulliford	2017	UK	usual care	models individual weight management intervention	usual care	Gulliford	[34]
Huse	2020	Australia/New Zealand	hypothetical intervention	models population obesity reduction impact	SSB promotion ban	ACE-Obesity Policy model	[35]
Kao	2020	N America	hypothetical intervention	models population obesity reduction impact	SSB tax	ACE-Prevention	[36]

Kent	2019	UK	intervention	models individual weight management intervention	weight loss programme	PRIMEtime-CE	[37]
Kianmehr	2022	N America	hypothetical change	models population obesity reduction impact	models impact of weight loss	BRAVO (Building, Relating, Assessing, and Validating Outcomes) diabetes microsimulation model	[38]
Kingston	2021	Australia/New Zealand	no change, no intervention	models population obesity impact	models impact of obesity	Kingston	[39]
Lal	2020	Australia/New Zealand	hypothetical change	models population obesity reduction impact	dietary change	ACE-Obesity Policy model	[40]
Lal	2017	Australia/New Zealand	hypothetical intervention	models population obesity reduction impact	SSB tax	CRE-Obesity model based on the Assessing Cost-Effectiveness in Prevention (ACE-Prevention) obesity model	[41]
Laxy	2020	N America	hypothetical intervention	models individual weight management intervention	health improvement/prevention intervention	CDC-RTI diabetes computer simulation model	[42]
Liu	2022	N America	hypothetical intervention	models population obesity reduction impact	SSB tax	Liu	[43]
Liu	2020	N America	intervention	models population obesity reduction impact	food labelling	CVD-PREDICT (Cardiovascular Disease Policy Model for Risk, Events, Detection, Interventions, Costs, and Trends) model	[44]
Long	2019	N America	hypothetical intervention	models population obesity reduction impact	SSB tax	CHOICES (Childhood Obesity Intervention Cost-Effectiveness Study) model	[45]
Long	2015	N America	hypothetical intervention	models population obesity reduction impact	SSB tax	ACE-Obesity Policy model	[46]
Lymer	2018	Australia/New Zealand	intervention	models individual weight management intervention	weight loss programme	NCDMod	[47]

Mantilla-Herrera	2018	Australia/New Zealand	hypothetical intervention	models population obesity reduction impact	food labelling	CRE-Obesity model based on the Assessing Cost-Effectiveness in Prevention (ACE-Prevention) obesity model	[48]
Michaud	2017	N America	intervention	models individual weight management intervention	weight loss programme	Michaud	[49]
Mytton	2018	UK	intervention	models individual weight management intervention	health improvement/prevention intervention	Mytton	[50]
Neumann	2016	Europe (non-UK)	hypothetical intervention	models individual weight management intervention	health improvement/prevention intervention	Neumann	[51]
Nomaguchi	2017	Australia/New Zealand	hypothetical intervention	models population obesity reduction impact	SSB tax	ACE-Prevention	[52]
Nuijten	2018	N America	intervention	models individual weight management intervention	weight loss programme	Nuijten	[53]
Pitt	2020	N America	hypothetical intervention	models population obesity reduction impact	meat price increase	Pitt	[54]
Robinson	2020	Australia/New Zealand	hypothetical intervention	models population obesity reduction impact	alcohol pricing	ACE-Obesity Policy model	[55]
Rognoni	2020	Europe (non-UK)	intervention	models individual weight management intervention	weight loss programme	Rognoni	[56]
Sanchez-Romero	2016	Other	hypothetical change	models population obesity reduction impact	sugar reduction	The Cardiovascular Disease (BMI) Policy model	[57]
Schell	2020	N America	no change, no intervention	models population obesity impact	models impact of obesity	Schell	[58]

Shangguan	2021	N America	hypothetical change	models population obesity reduction impact	sugar reduction	CVD-PREDICT (Cardiovascular Disease Policy Model for Risk, Events, Detection, Interventions, Costs, and Trends) model	[59]
Smith	2016	N America	intervention	models individual weight management intervention	health improvement/prevention intervention	Smith	[60]
Sonntag	2017	Europe (non-UK)	no change, no intervention	models population obesity impact	models impact of obesity	DC-Obesity	[61]
Springmann	2016	Global	hypothetical change	models population obesity reduction impact	dietary change	Springmann	[62]
Su	2018	N America	hypothetical change	models population obesity reduction impact	models impact of weight loss	Dall	[63]
Su	2016	N America	intervention	models individual weight management intervention	health improvement/prevention intervention	Dall	[64]
Su	2015	N America	no change, no intervention	models population obesity impact	models impact of obesity	Dall	[65]
Thomas	2017	UK	intervention	models individual weight management intervention	health improvement/prevention intervention	School for Public Health Research Diabetes Prevention Model.	[66]
Thomas	2022	UK	intervention	models population obesity reduction impact	advertising ban	School for Public Health Research Diabetes Prevention Model.	[67]
Veerman	2016	Australia/New Zealand	hypothetical intervention	models population obesity reduction impact	SSB tax	ACE-Prevention	[68]
Verhaeghe	2016	Europe (non-UK)	hypothetical change	models population obesity reduction impact	models impact of weight loss	Verhaeghe	[69]

Vreman	2017	N America	hypothetical change	models population obesity reduction impact	sugar reduction	Vreman	[70]
Walter	2022	Europe (non-UK)	usual care	models individual weight management intervention	usual care	Walter	[71]
Wilde	2019	N America	hypothetical intervention	models population obesity reduction impact	SSB tax	CVD-PREDICT (Cardiovascular Disease Policy Model for Risk, Events, Detection, Interventions, Costs, and Trends) model	[72]
Willems	2020	Europe (non-UK)	intervention	models individual weight management intervention	health improvement/prevention intervention	ToyBOX study model	[73]
Wilson	2015	N America	intervention	models individual weight management intervention	weight loss programme	Archimedes model	[74]
Zomer	2016	UK	hypothetical change	models population obesity reduction impact	models impact of weight loss	Zomer	[75]

Table S5: Actual and hypothetical interventions modelled

Intervention	Examples	n (%)	of which were hypothetical
**Taxation or subsidy**	Sugar-sweetened beverage tax, vegetable subsidy	16 (27%)	15 (94%)
**Individual weight management intervention**	In person or digitally delivered group weight management course. Includes usual care.	15 (26%)	2 (13%)
**Health improvement or prevention intervention**	Diabetes prevention programme, mass healthy lifestyle campaign	12 (21%)	3 (25%)
**Regulation**	Food labelling, sugar-sweetened beverage reformulation	7 (12%)	5 (72%)
**Population dietary change**	Achievement of government dietary guidelines or sugar reduction strategy	5 (9%)	5 (100%)
**Active transport**	Policy introduction to support active transport use	3 (5%)	3 (100%)
	TOTAL	58	

Table S6: Frequency of model use

Model name	Frequency of use	% of included studies
ACE-Obesity (Assessing Cost-Effectiveness in Obesity) Policy model	10	14%
School for Public Health Research Diabetes Prevention model	5	7%
ACE-Prevention (Assessing Cost-Effectiveness in Prevention) model	4	6%
Dall	4	6%
CVD-PREDICT (Cardiovascular Disease Policy Model for Risk, Events, Detection, Interventions, Costs, and Trends) model	3	4%
UK Health Forum model	3	4%
Brown	2	3%
Chen	2	3%
CHOICES (Childhood Obesity Intervention Cost-Effectiveness Study) model	2	3%
CRE-Obesity model	2	3%
PRIMEtime-CE	2	3%
The Cardiovascular Disease (BMI) Policy model	2	3%
Models used once only	32	44% (1% each)

Table S7: Table of the models included in the scoping review

Model name	Health economic measure(s)	Model type	Event simulation approach	Time horizon(s)	Validation	Reference
ACE-Obesity Policy model	HALYs<br>QALYs	Multistate life tables	Risk Equation / Change in Risk Factors	Lifetime	Y	[7]
ACE-Prevention	DALYs<br>HALYs	Multistate life tables	Risk Equation / Change in Risk Factors	Lifetime	Y	[76]
An	Direct BMI to cost calculation	Microsimulation	BMI Function / Change in BMI	Short term, Long term	N	[6]
Archimedes model	QALYs	Microsimulation	Risk Equation / Change in Risk Factors	Short term, Long term	Y	[77]
Arrospide	QALYs	Discrete event simulation	Disease Incidence Estimate /BMI Group related RR	Lifetime	Y	[10]
Basu	QALYs	Microsimulation	Others / Others	Long term, lifetime	N	[13]
Bjornelv	Direct BMI to cost calculation	Markov models without interaction	BMI Group Function / Change in BMI Group	Short term, Long term, Lifetime	N	[15]
Blakely	HALYs	Multistate life tables	Risk Equation / Change in Risk Factors	Lifetime	Y	[16]
BODE (Burden of Disease Epidemiology, Equity and Cost-Effectiveness) Programme model	QALYs	Multistate life tables	Risk Equation / Change in Risk Factors	Lifetime	Y	[78]
BRAVO (Building, Relating, Assessing, and Validating Outcomes) diabetes microsimulation model	QALYs	Microsimulation	Risk Equation / Change in Risk Factors	Short term, Long term, Lifetime	Y	[79]
Brown	HALYs	Multistate life tables	Disease Incidence Estimate / BMI related relative risk (RR)	Lifetime	N	[20]
CDC-RTI diabetes computer simulation model	QALYs	Markov chain models and Markov individual event history models	Risk Equation / Change in Risk Factors	Lifetime	Y	[80]
Chen	Direct health status to cost calculation	Microsimulation	Risk Equation / Change in Risk Factors	Short term, Long term	N	[24]

Choi	QALYs	Microsimulation	Risk Equation / Change in Risk Factors	Lifetime	Y	[25]
CHOICES (Childhood Obesity Intervention Cost-Effectiveness Study) model	Direct BMI to cost calculation	Microsimulation	BMI Function / Change in BMI	Short term, Long term	N	[81]
CRE-Obesity model	HALYs	Multistate life tables	Risk Equation / Change in Risk Factors	Long term, lifetime	N	[41]
CVD-PREDICT (Cardiovascular Disease Policy Model for Risk, Events, Detection, Interventions, Costs, and Trends) model	QALYs	Microsimulation	Risk Equation / Change in Risk Factors	Short term, lifetime	Y	[82]
Dall	Direct health status to cost calculation	Microsimulation	Risk Equation / Change in Risk Factors	Lifetime	Y	[65]
DC (Differential Costs)-Obesity	Direct BMI to cost calculation	Markov chain models and Markov individual event history models	Risk Equation / Change in Risk Factors	Lifetime	N	[61]
Fallah-Fini	QALYs	Markov models without interaction	Risk Equation / Change in Risk Factors	Lifetime	N	[30]
Galvain	QALYs	Markov models without interaction	Risk Equation / Change in Risk Factors	Lifetime	N	[31]
Gulliford	QALYs	Markov models without interaction	Risk Equation / Change in Risk Factors	Lifetime	N	[34]
Kingston	only uses DFLE (uncosted)	Markov chain models and Markov individual event history models	Disease Incidence Estimate /Obesity related RR	Lifetime	N	[39]
Liu	DALYs	Multistate life tables	Risk Equation / Change in Risk Factors	Lifetime	N	[43]
Michaud	Direct health status to cost calculation	Markov models without interaction	Disease Incidence Estimate / BMI related relative risk (RR)	Lifetime	N	[49]
Mytton	QALYs	Microsimulation	Risk Equation / Change in Risk Factors	Lifetime	Y	[83]
NCDMod	Direct health status to cost calculation	Microsimulation	Risk Equation / Change in Risk Factors	Long term	Y	[84]

Neumann	QALYs	Markov models without interaction	Risk Equation / Change in Risk Factors	Lifetime	N	[51]
Nuijten	Direct health status to cost calculation	Decision tree	Disease Incidence Estimate /Obesity related RR	Short term, Long term	Y	[53]
OECD SPHeP-NCD (Strategic Public Health Planning for NCDs) model	Mortality and years lived in good health	Microsimulation	Risk Equation / Change in Risk Factors	Long term	N	[85]
Pitt	QALYs	Microsimulation	BMI Function / Change in BMI	Long term	N	[54]
PRIMEtime-CE	QALYs	Multistate life tables	Risk Equation / Change in Risk Factors	Short term, Long term, Lifetime	Y	[86]
Rognoni	QALYs	Markov models without interaction	Risk Equation / Change in Risk Factors	Lifetime	Y	[56]
Schell	Direct BMI to cost calculation	Markov models without interaction	BMI Group Function / Change in BMI Group	Lifetime	N	[58]
School for Public Health Research Diabetes Prevention Model	QALYs	Microsimulation	Risk Equation / Change in Risk Factors	Long term, lifetime	Y	[76]
Smith	QALYs	Markov models without interaction	Risk Equation / Change in Risk Factors	Short term	N	[60]
Springmann	Direct health status to cost calculation	Comparative risk assessment	Risk Equation / Change in Risk Factors	Long term	N	[62]
The Cardiovascular Disease (BMI) Policy model	QALYs	Microsimulation	Risk Equation / Change in Risk Factors	Long term, lifetime	Y	[33]
ToyBOX study model	QALYs	Markov models without interaction	Disease Incidence Estimate /BMI Group related RR	Long Term	N	[87]
UK Health Forum	QALYs	Microsimulation	Risk Equation / Change in Risk Factors	Long term, lifetime	Y	[11]
Verhaeghe	QALYs	Markov models without interaction	BMI Function / Change in BMI	Long Term	N	[69]
Vreman	DALYs	Microsimulation	Risk Equation / Change in Risk Factors	Long Term	Y	[70]
Walter	QALYs	Microsimulation	Risk Equation / Change in Risk Factors	Long Term	N	[71]

Zomer	QALYs	Markov models without interaction	Risk Equation / Change in Risk Factors	Long Term	N	[75]

References for Supporting Information Tables

1. McLaughlin J, Sillero-Rejon C, Moore T, McLeod H. Study protocol: Health Economic Models for Estimating the Benefits of Obesity Reduction and Weight Loss: a Scoping Review. The Open Science Framework Registry. doi:https://doi.org/10.17605/OSF.IO/4U53Y

2. Schwander B, Nuijten M, Hiligsmann M, et al. Identification and expert panel rating of key structural approaches applied in health economic obesity models. *Health Policy Technol*. 2020;9(3):314-322. doi:10.1016/j.hlpt.2020.03.005

3. Ahern AL, Breeze P, Fusco F, et al. Effectiveness and cost-effectiveness of referral to a commercial open group behavioural weight management programme in adults with overweight and obesity: 5-year follow-up of the WRAP randomised controlled trial. *Lancet Public Health*. 2022;7(10):e866-e875. doi:10.1016/S2468-2667(22)00226-2

4. Ahern AL, Wheeler GM, Aveyard P, et al. Extended and standard duration weight-loss programme referrals for adults in primary care (WRAP): a randomised controlled trial. *The Lancet*. 2017;389(10085):2214-2225. doi:10.1016/S0140-6736(17)30647-5

5. Amies-Cull B, Briggs ADM, Scarborough P. Estimating the potential impact of the UK government's sugar reduction programme on child and adult health: Modelling study. *The BMJ*. 2019;365:l1417. doi:10.1136/bmj.l1417

6. An R, Zheng J, Xiang X. Projecting the Influence of Sugar-Sweetened Beverage Warning Labels and Restaurant Menu Labeling Regulations on Energy Intake, Weight Status, and Health Care Expenditures in US Adults: A Microsimulation. *J Acad Nutr Diet*. 2022;122(2):334-344. doi:10.1016/j.jand.2021.05.006

7. Ananthapavan J, Sacks G, Brown V, et al. Priority-setting for obesity prevention—The Assessing Cost-Effectiveness of obesity prevention policies in Australia (ACE-Obesity Policy) study. *PLoS One*. 2020;15(6):e0234804. doi:10.1371/journal.pone.0234804

8. Ananthapavan J, Tran HNQ, Morley B, et al. Cost-effectiveness of LiveLighter® - a mass media public education campaign for obesity prevention. *PLoS One*. 2022;17(9):e0274917. doi:10.1371/journal.pone.0274917

9. Ananthapavan J, Sacks G, Orellana L, et al. Cost–Benefit and Cost–Utility Analyses to Demonstrate the Potential Value-for-Money of Supermarket Shelf Tags Promoting Healthier Packaged Products in Australia. *Nutrients*. 2022;14(9):1919. doi:10.3390/nu14091919

10. Arrospide A, Ibarrondo O, Castilla I, Larrañaga I, Mar J. Development and Validation of a Discrete Event Simulation Model to Evaluate the Cardiovascular Impact of Population Policies for Obesity. *Medical Decision Making*. 2022;42(2):241-254. doi:10.1177/0272989X211032964

11. Avenell A, Robertson C, Skea Z, et al. Bariatric surgery, lifestyle interventions and orlistat for severe obesity: The rebalance mixed-methods systematic review and economic evaluation. *Health Technol Assess (Rockv)*. 2018;22(68):1-246. doi:10.3310/hta22680

12. Basto-Abreu A, Barrientos-Gutiérrez T, Vidaña-Pérez D, et al. Cost-effectiveness of the sugar-sweetened beverage excise tax in Mexico. *Health Aff*. 2019;38(11):1824-1831. doi:10.1377/hlthaff.2018.05469

13. Basu S, Jacobs LM, Epel E, Schillinger D, Schmidt L. Cost-effectiveness of a workplace ban on sugar-sweetened beverage sales: A microsimulation model. *Health Aff*. 2020;39(7):1140-1148. doi:10.1377/hlthaff.2019.01483

14. Bates SE, Thomas C, Islam N, et al. Using health economic modelling to inform the design and development of an intervention: estimating the justifiable cost of weight loss maintenance in the UK. *BMC Public Health*. 2022;22:290. doi:10.1186/s12889-022-12737-5

15. Bjørnelv GMW, Halsteinli V, Kulseng BE, Sonntag D, Ødegaard RA. Modeling Obesity in Norway (The MOON Study): A Decision-Analytic Approach—Prevalence, Costs, and Years of Life Lost. *Medical Decision Making*. 2021;41(1):21-36. doi:10.1177/0272989X20971589

16. Blakely T, Cleghorn C, Mizdrak A, et al. The effect of food taxes and subsidies on population health and health costs: a modelling study. *Lancet Public Health*. 2020;5(7):e404-e413. doi:10.1016/S2468-2667(20)30116-X

17. Bourke EJ, Veerman JL. The potential impact of taxing sugar drinks on health inequality in Indonesia. *BMJ Glob Health*. 2018;3:e000923. doi:10.1136/bmjgh-2018-000923

18. Boyers D, Retat L, Jacobsen E, et al. Cost-effectiveness of bariatric surgery and non-surgical weight management programmes for adults with severe obesity: a decision analysis model. *Int J Obes*. 2021;45(10):2179-2190. doi:10.1038/s41366-021-00849-8

19. Breeze PR, Thomas C, Squires H, et al. Cost-effectiveness of population-based, community, workplace and individual policies for diabetes prevention in the UK. *Diabetic Medicine*. 2017;34(8):1136-1144. doi:10.1111/dme.13349

20. Brown V, Moodie M, Cobiac L, Mantilla H, Carter R. Obesity-related health impacts of active transport policies in Australia – a policy review and health impact modelling study. *Aust N Z J Public Health*. 2017;41(6):611-616. doi:10.1111/1753-6405.12726

21. Brown V, Moodie M, Cobiac L, Mantilla Herrera AM, Carter R. Obesity-related health impacts of fuel excise taxation- an evidence review and cost-effectiveness study. *BMC Public Health*. 2017;17:359. doi:10.1186/s12889-017-4271-2

22. Brown V, Moodie M, Mantilla Herrera AM, Veerman JL, Carter R. Active transport and obesity prevention – A transportation sector obesity impact scoping review and assessment for Melbourne, Australia. *Prev Med (Baltim)*. 2017;96:49-66. doi:10.1016/j.ypmed.2016.12.020

23. Chen F, Su W, Becker SH, et al. Clinical and economic impact of a digital, remotely-delivered intensive behavioral counseling program on medicare beneficiaries at risk for diabetes and cardiovascular disease. *PLoS One*. 2016;11(10):e0163627. doi:10.1371/journal.pone.0163627

24. Chen F, Jasik CB, Dall TM, Siego CV. Impact of a Digitally Enhanced Diabetes Self-Management Program on Glycemia and Medical Costs. *Science of Diabetes Self-Management and Care*. 2022;48(4):258-269. doi:10.1177/26350106221100779

25. Choi SE, Seligman H, Basu S. Cost Effectiveness of Subsidizing Fruit and Vegetable Purchases Through the Supplemental Nutrition Assistance Program. *Am J Prev Med*. 2017;52(5):e147-e155. doi:10.1016/j.amepre.2016.12.013

26. Cleghorn C, Wilson N, Nair N, et al. Health benefits and cost-effectiveness from promoting smartphone apps for weight loss: Multistate life table modeling. *JMIR MHealth UHealth*. 2019;7(1):e11118. doi:10.2196/11118

27. Cobiac LJ, Tam K, Veerman L, Blakely T. Taxes and Subsidies for Improving Diet and Population Health in Australia: A Cost-Effectiveness Modelling Study. *PLoS Med*. 2017;14(2):e1002232. doi:10.1371/journal.pmed.1002232

28. Crino M, Herrera AMM, Ananthapavan J, et al. Modelled cost-effectiveness of a package size cap and a kilojoule reduction intervention to reduce energy intake from sugar-sweetened beverages in Australia. *Nutrients*. 2017;9(9):1-17. doi:10.3390/nu9090983

29. Dall TM, Storm M V., Semilla AP, Wintfeld N, O'Grady M, Venkat Narayan KM. Value of lifestyle intervention to prevent diabetes and sequelae. *Am J Prev Med*. 2015;48(3):271-280. doi:10.1016/j.amepre.2014.10.003

30. Fallah-Fini S, Adam A, Cheskin LJ, Bartsch SM, Lee BY. The Additional Costs and Health Effects of a Patient Having Overweight or Obesity: A Computational Model. *Obesity*. 2017;25(10):1809-1815. doi:10.1002/oby.21965

31. Galvain T, Patel S, Kabiri M, Tien S, Casali G, Pournaras DJ. Cost-effectiveness of bariatric and metabolic surgery, and implications of COVID-19 in the United Kingdom. *Surgery for Obesity and Related Diseases*. 2021;17(11):1897-1904. doi:10.1016/j.soard.2021.07.009

32. Goryakin Y, Aldea A, Lerouge A, et al. Promoting sport and physical activity in Italy: A costeffectiveness analysis of seven innovative public health policies. *Ann Ig*. 2019;31(6):614-625. doi:10.7416/ai.2019.2321

33. Gray CM, Wyke S, Zhang R, et al. Long-term weight loss following a randomised controlled trial of a weight management programme for men delivered through professional football clubs: the Football Fans in Training follow-up study. *Public Health Research*. 2018;6(9):1-114. doi:10.3310/phr06090

34. Gulliford MC, Charlton J, Prevost T, et al. Costs and Outcomes of Increasing Access to Bariatric Surgery: Cohort Study and Cost-Effectiveness Analysis Using Electronic Health Records. *Value in Health*. 2017;20(1):85-92. doi:10.1016/j.jval.2016.08.734

35. Huse O, Ananthapavan J, Sacks G, et al. The potential cost-effectiveness of mandatory restrictions on price promotions for sugar-sweetened beverages in Australia. *Int J Obes*. 2020;44(5):1011-1020. doi:10.1038/s41366-019-0495-9

36. Kao KE, Jones AC, Ohinmaa A, Paulden M. The health and financial impacts of a sugary drink tax across different income groups in Canada. *Econ Hum Biol*. 2020;38:100869. doi:10.1016/j.ehb.2020.100869

37. Kent S, Aveyard P, Astbury N, Mihaylova B, Jebb SA. Is Doctor Referral to a Low-Energy Total Diet Replacement Program Cost-Effective for the Routine Treatment of Obesity? *Obesity*. 2019;27(3):391-398. doi:10.1002/oby.22407

38. Kianmehr H, Zhang P, Luo J, et al. Potential Gains in Life Expectancy Associated with Achieving Treatment Goals in US Adults with Type 2 Diabetes. *JAMA Netw Open*. Published online 2022:E227705. doi:10.1001/jamanetworkopen.2022.7705

39. Kingston A, Byles J, Kiely K, Anstey KJ, Jagger C. The Impact of Smoking and Obesity on Disability-Free Life Expectancy in Older Australians. *J Gerontol A Biol Sci Med Sci*. 2021;76(7):1265-1272. doi:10.1093/gerona/glaa290

40. Lal A, Peeters A, Brown V, et al. The modelled population obesity-related health benefits of reducing consumption of discretionary foods in Australia. *Nutrients*. 2020;12:649. doi:10.3390/nu12030649

41. Lal A, Mantilla-Herrera AM, Veerman L, et al. Modelled health benefits of a sugar-sweetened beverage tax across different socioeconomic groups in Australia: A cost-effectiveness and equity analysis. *PLoS Med*. 2017;14(6):e1002326. doi:10.1371/journal.pmed.1002326

42. Laxy M, Zhang P, Ng BP, et al. Implementing Lifestyle Change Interventions to Prevent Type 2 Diabetes in US Medicaid Programs: Cost Effectiveness, and Cost, Health, and Health Equity Impact. *Appl Health Econ Health Policy*. 2020;18(5):713-726. doi:10.1007/s40258-020-00565-w

43. Liu S, Veugelers PJ, Maximova K, Ohinmaa A. Modelling the health and economic impact of sugary sweetened beverage tax in Canada. *PLoS One*. 2022;17(11):e0277306. doi:10.1371/journal.pone.0277306

44. Liu J, Mozaffarian D, Sy S, et al. Health and Economic Impacts of the National Menu Calorie Labeling Law in the United States: A Microsimulation Study. *Circ Cardiovasc Qual Outcomes*. 2020;13(6):E006313. doi:10.1161/CIRCOUTCOMES.119.006313

45. Long MW, Polacsek M, Bruno P, et al. Cost-Effectiveness Analysis and Stakeholder Evaluation of 2 Obesity Prevention Policies in Maine, US. *J Nutr Educ Behav*. 2019;51(10):1177-1187. doi:10.1016/j.jneb.2019.07.005

46. Long MW, Gortmaker SL, Ward ZJ, et al. Cost Effectiveness of a Sugar-Sweetened Beverage Excise Tax in the U.S. *Am J Prev Med*. 2015;49(1):112-123. doi:10.1016/j.amepre.2015.03.004

47. Lymer S, Schofield D, Cunich M, et al. The Population Cost-Effectiveness of Weight Watchers with General Practitioner Referral Compared with Standard Care. *Obesity*. 2018;26(8):1261-1269. doi:10.1002/oby.22216

48. Mantilla-Herrera AM, Crino M, Erskine HE, et al. Cost-effectiveness of product reformulation in response to the health star rating food labelling system in Australia. *Nutrients*. 2018;10:614. doi:10.3390/nu10050614

49. Michaud TL, You W, Wilson KE, et al. Cost effectiveness and return on investment of a scalable community weight loss intervention. *Prev Med (Baltim)*. 2017;105:295-303. doi:10.1016/j.ypmed.2017.10.011

50. Mytton OT, Jackson C, Steinacher A, et al. The current and potential health benefits of the National Health Service Health Check cardiovascular disease prevention programme in England: A microsimulation study. *PLoS Med*. 2018;15(3):e1002517. doi:10.1371/journal.pmed.1002517

51. Neumann A, Lindholm L, Norberg M, Schoffer O, Klug SJ, Norström F. The cost-effectiveness of interventions targeting lifestyle change for the prevention of diabetes in a Swedish primary care and community based prevention program. *European Journal of Health Economics*. 2017;18(7):905-919. doi:10.1007/s10198-016-0851-9

52. Nomaguchi T, Cunich M, Zapata-Diomedi B, Veerman JL. The impact on productivity of a hypothetical tax on sugar-sweetened beverages. *Health Policy (New York)*. 2017;121(6):715-725. doi:10.1016/j.healthpol.2017.04.001

53. Nuijten M, Marczewska A, Araujo Torres K, Rasouli B, Perugini M. A health economic model to assess the cost-effectiveness of OPTIFAST for the treatment of obesity in the United States. *J Med Econ*. 2018;21(9):835-844. doi:10.1080/13696998.2018.1468334

54. Pitt AL, Goldhaber-Fiebert JD, Brandeau ML. Public Health Interventions with Harms and Benefits: A Graphical Framework for Evaluating Tradeoffs. *Medical Decision Making*. 2020;40(8):978-989. doi:10.1177/0272989X20960458

55. Robinson E, Nguyen P, Jiang H, et al. Increasing the price of alcohol as an obesity prevention measure: The potential cost-effectiveness of introducing a uniform volumetric tax and a minimum floor price on alcohol in Australia. *Nutrients*. 2020;12:603. doi:10.3390/nu12030603

56. Rognoni C, Armeni P, Tarricone R, Donin G. Cost–benefit Analysis in Health Care: The Case of Bariatric Surgery Compared With Diet. *Clin Ther*. 2020;42(1):60-75.e7. doi:10.1016/j.clinthera.2019.12.001

57. Sánchez-Romero LM, Penko J, Coxson PG, et al. Projected Impact of Mexico's Sugar-Sweetened Beverage Tax Policy on Diabetes and Cardiovascular Disease: A Modeling Study. *PLoS Med*. 2016;13(11):e1002158. doi:10.1371/journal.pmed.1002158

58. Schell RC, Just DR, Levitsky DA. Predicted Lifetime Third-Party Costs of Obesity for Black and White Adolescents with Race-Specific Age-Related Weight Gain. *Obesity*. 2020;28(2):397-403. doi:10.1002/oby.22690

59. Shangguan S, Mozaffarian D, Sy S, et al. Health Impact and Cost-Effectiveness of Achieving the National Salt and Sugar Reduction Initiative Voluntary Sugar Reduction Targets in the United States: A Microsimulation Study. *Circulation*. 2021;144(17):1362-1376. doi:10.1161/CIRCULATIONAHA.121.053678

60. Smith KJ, Kuo S, Zgibor JC, et al. Cost effectiveness of an internet-delivered lifestyle intervention in primary care patients with high cardiovascular risk. *Prev Med (Baltim)*. 2016;87:103-109. doi:10.1016/j.ypmed.2016.02.036

61. Sonntag D, Jarczok MN, Ali S. DC-Obesity: A New Model for Estimating Differential Lifetime Costs of Overweight and Obesity by Socioeconomic Status. *Obesity*. 2017;25(9):1603-1609. doi:10.1002/oby.21913

62. Springmann M, Charles H, Godfray J, Rayner M, Scarborough P. Analysis and valuation of the health and climate change cobenefits of dietary change. *PNAS*. 2016;113(15):4146-4151. doi:10.5287/bodleian:XObxm2ebO

63. Su W, Chen F, Dall TM, Zvenyach T, Kyle TK, Perreault L. Where can obesity management policy make the largest impact? Evaluating sub-populations through a microsimulation approach. *J Med Econ*. 2018;21(9):936-943. doi:10.1080/13696998.2018.1496922

64. Su WW, Chen F, Dall TM, Iacobucci W, Perreault L. Return on investment for digital behavioral counseling in patients with prediabetes and cardiovascular disease. *Prev Chronic Dis*. 2016;13:150357. doi:10.5888/pcd13.150357

65. Su W, Huang J, Chen F, et al. Modeling the clinical and economic implications of obesity using microsimulation. *J Med Econ*. 2015;18(11):886-897. doi:10.3111/13696998.2015.1058805

66. Thomas C, Sadler S, Breeze P, Squires H, Gillett M, Brennan A. Assessing the potential return on investment of the proposed UK NHS diabetes prevention programme in different population subgroups: An economic evaluation. *BMJ Open*. 2017;7:e014953. doi:10.1136/bmjopen-2016-014953

67. Thomas C, Breeze P, Cummins S, Cornelsen L, Yau A, Brennan A. The health, cost and equity impacts of restrictions on the advertisement of high fat, salt and sugar products across the transport for London network: a health economic modelling study. *Int J Behav Nutr Phys Act*. 2022;19:93. doi:10.1186/s12966-022-01331-y

68. Veerman JL, Sacks G, Antonopoulos N, Martin J. The impact of a tax on sugar-sweetened beverages on health and health care costs: A modelling study. *PLoS One*. 2016;11(4):e0151460. doi:10.1371/journal.pone.0151460

69. Verhaeghe N, De Greve O, Annemans L. The potential health and economic effect of a Body Mass Index decrease in the overweight and obese population in Belgium. *Public Health*. 2016;134:26-33. doi:10.1016/j.puhe.2016.01.015

70. Vreman RA, Goodell AJ, Rodriguez LA, Porco TC, Lustig RH, Kahn JG. Health and economic benefits of reducing sugar intake in the USA, including effects via non-alcoholic fatty liver disease: A microsimulation model. *BMJ Open*. 2017;7:e013543. doi:10.1136/bmjopen-2016-013543

71. Walter E, Langer FB, Beckerhinn P, Hoffer F, Prager G. Impact of metabolic surgery on cost and long-term health outcome: a cost-effectiveness approach. *Surgery for Obesity and Related Diseases*. 2022;18(2):260-270. doi:10.1016/j.soard.2021.10.012

72. Wilde P, Huang Y, Sy S, et al. Cost-effectiveness of a US national sugar-sweetened beverage tax with a multistakeholder approach: Who pays and who benefits. *Am J Public Health*. 2019;109(2):276-284. doi:10.2105/AJPH.2018.304803

73. Willems R, Pil L, Lambrinou CP, et al. Methodology of the health economic evaluation of the Feel4Diabetes-study. *BMC Endocr Disord*. 2020;20(Suppl 1):14. doi:10.1186/s12902-019-0471-3

74. Wilson KJ, Brown HS, Bastida E. Cost-Effectiveness of a Community-Based Weight Control Intervention Targeting a Low-Socioeconomic-Status Mexican-Origin Population. *Health Promot Pract*. 2015;16(1):101-108. doi:10.1177/1524839914537274

75. Zomer E, Leach R, Trimmer C, et al. Effectiveness and cost-effectiveness of interventions that cause weight loss and reduce the risk of cardiovascular disease. *Diabetes Obes Metab*. 2017;19(1):118-124. doi:10.1111/dom.12792

76. Breeze P, Thomas C, Squires H, et al. *School for Public Health Research (SPHR) Diabetes Prevention Model: Detailed Description of Model Background, Methods, Assumptions and Parameters*.; 2015. Accessed September 21, 2023. https://eprints.whiterose.ac.uk/97829/1/Breeze%20et%20al_SPHR%20Diabetes%20Prevention%20Model.pdf

77. Schlessinger L, Eddy DM. Archimedes: a new model for simulating health care systems-the mathematical formulation. *J Biomed Inform*. 2002;1:37-50. doi:doi: 10.1016/s1532-0464(02)00006-0.

78. Cleghorn C, Blakely T, Nghiem N, Mizdrak A, Wilson N. *Technical Report for BODE 3 Diet Intervention and Multistate Lifetable Models Burden of Disease Epidemiology, Equity and Cost-Effectiveness Programme (BODE 3) Technical Report: Number 16*.; 2017. Accessed September 21, 2023. https://www.otago.ac.nz/__data/assets/pdf_file/0015/330009/technical-report-for-bode-diet-intervention-and-multistate-lifetable-models-670797.pdf

79. Shao H, Fonseca V, Stoecker C, Liu S, Shi L. Novel Risk Engine for Diabetes Progression and Mortality in USA: Building, Relating, Assessing, and Validating Outcomes (BRAVO). *Pharmacoeconomics*. 2018;36(9):1125-1134. doi:10.1007/s40273-018-0662-1

80. The CDC Diabetes Cost-effectiveness Group. Cost-effectiveness of Intensive Glycemic Control, Intensified Hypertension Control, and Serum Cholesterol Level Reduction for Type 2 Diabetes. *JAMA*. 2002;287(10):2542-2551.

81. Gortmaker SL, Wang YC, Long MW, et al. Three interventions that reduce childhood obesity are projected to save more than they cost to implement. *Health Aff*. 2015;34(11):1932-1939. doi:10.1377/hlthaff.2015.0631

82. Pandya A, Sy S, Cho S, Alam S, Weinstein MC, Gaziano TA. Validation of a Cardiovascular Disease Policy Microsimulation Model Using Both Survival and Receiver Operating Characteristic Curves. *Medical Decision Making*. 2017;37(7):802-814. doi:10.1177/0272989X17706081

83. Briggs ADM, Mytton OT, Kehlbacher A, et al. Health impact assessment of the UK soft drinks industry levy: a comparative risk assessment modelling study. *Lancet Public Health*. 2017;2(1):e15-e22. doi:10.1016/S2468-2667(16)30037-8

84. Lymer S, Schofield D, Lee CMY, Colagiuri S. NCDMod: A Microsimulation Model Projecting Chronic Disease and Risk Factors for Australian Adults. *Int J Microsimul*. 2016;9(3):103-139.

85. Devaux M, Lerouge A, Ventelou B, et al. Assessing the potential outcomes of achieving the World Health Organization global non-communicable diseases targets for risk factors by 2025: is there also an economic dividend? *Public Health*. 2019;169:173-179. doi:10.1016/j.puhe.2019.02.009

86. Briggs ADM, Cobiac LJ, Wolstenholme J, Scarborough P. PRIMEtime CE: A multistate life table model for estimating the cost-effectiveness of interventions affecting diet and physical activity. *BMC Health Serv Res*. 2019;19:485. doi:10.1186/s12913-019-4237-4

87. Pil L, Putman K, Cardon G, et al. Establishing a method to estimate the cost-effectiveness of a kindergarten-based, family-involved intervention to prevent obesity in early childhood. The ToyBox-study. *Obesity Reviews*. 2014;15(SUPPL.3):81-89. doi:10.1111/obr.12179
